# Manipulation of hot carrier cooling dynamics in two-dimensional Dion–Jacobson hybrid perovskites via Rashba band splitting

**DOI:** 10.1038/s41467-021-24258-7

**Published:** 2021-06-28

**Authors:** Jun Yin, Rounak Naphade, Partha Maity, Luis Gutiérrez-Arzaluz, Dhaifallah Almalawi, Iman S. Roqan, Jean-Luc Brédas, Osman M. Bakr, Omar F. Mohammed

**Affiliations:** 1 Advanced Membranes and Porous Materials Center, Division of Physical Science and Engineering, King Abdullah University of Science and Technology, Thuwal, 23955-6900 Kingdom of Saudi Arabia; 2 KAUST Catalysis Center, Division of Physical Sciences and Engineering, King Abdullah University of Science and Technology, Thuwal, 23955-6900 Kingdom of Saudi Arabia; 3 Division of Physical Sciences and Engineering, King Abdullah University of Science and Technology, Thuwal, 23955-6900 Kingdom of Saudi Arabia; 4 Department of Physics, College of science, Taif University, Taif, 21944 Saudi Arabia; 5 Department of Chemistry and Biochemistry, The University of Arizona, Tucson, 85721-0088 AZ USA

**Keywords:** Electronic properties and materials, Two-dimensional materials, Atomistic models, Optical spectroscopy

## Abstract

Hot-carrier cooling processes of perovskite materials are typically described by a single parabolic band model that includes the effects of carrier-phonon scattering, hot phonon bottleneck, and Auger heating. However, little is known (if anything) about the cooling processes in which the spin-degenerate parabolic band splits into two spin-polarized bands, i.e., the Rashba band splitting effect. Here, we investigated the hot-carrier cooling processes for two slightly different compositions of two-dimensional Dion–Jacobson hybrid perovskites, namely, (3AMP)PbI_4_ and (4AMP)PbI_4_ (3AMP = 3-(aminomethyl)piperidinium; 4AMP = 4-(aminomethyl)piperidinium), using a combination of ultrafast transient absorption spectroscopy and first-principles calculations. In (4AMP)PbI_4_, upon Rashba band splitting, the spin-dependent scattering of hot electrons is responsible for accelerating hot-carrier cooling at longer delays. Importantly, the hot-carrier cooling of (4AMP)PbI_4_ can be extended by manipulating the spin state of the hot carriers. Our findings suggest a new approach for prolonging hot-carrier cooling in hybrid perovskites, which is conducive to further improving the performance of hot-carrier-based optoelectronic and spintronic devices.

## Abstract (web summary)

Hybrid perovskite is a promising class of material for optoelectronic applications due to the slow hot-carrier cooling, yet the process is not well-understood in material with Rashba band splitting. Here, the authors reveal spin-flipping and spin-dependent scattering of hot electrons are responsible for accelerating the cooling at longer delays.

## Introduction

Two-dimensional (2D) hybrid perovskites are promising optoelectronic semiconductors because they offer greater structural diversity and stability compared to their three-dimensional (3D) counterparts^1–3^. In addition, the structural arrangement in 2D hybrid perovskites is akin to quantum wells (i.e., inorganic layers electronically isolated by insulating organic layers), giving rise to intriguing physical phenomena, such as quantum and dielectric confinement effects^4^, the optical Stark effect^5^, and coherent phonon interactions^6–8^. In addition, similar to 3D hybrid perovskites, the presence of heavy atoms (e.g., lead) can induce Rashba band splitting because of inversion symmetry breaking^9,10^, making such materials promising candidates for manipulating the spin states in spintronic devices. On the other hand, the spontaneous polarization properties of ferroelectric 2D perovskites can enhance free charge carrier generation and break the Shockley-Queisser limit for the bulk photovoltaic effect^11,12^.

There is also a growing interest in exploring applications involving hot-carrier-based optoelectronic and spintronic devices, especially hot-carrier solar cells (HCSCs), using 2D hybrid perovskites due to the deceleration of their hot-carrier cooling rate, which is mainly governed by quantum and dielectric confinement^8,13,14^. In this case, the hot-carrier cooling time can be extended up to tens of picoseconds via the combined effects of hot-phonon bottleneck^15^, enhanced Auger heating^16^, and formation of large polarons^17–19^. The cooling processes of hot carriers in perovskite materials are typically described using the single parabolic band model by assuming that the photogenerated hot carriers experience cooling via carrier-phonon scattering, optical phonon emission, acoustic-optical phonon upconversion, and thermal equilibrium^16,20^. However, when the spin degeneracy of the parabolic band is lifted and two spin-polarized band form, the cooling processes will be strongly influenced by having hot carriers with different spin states. Thus, understanding the mechanisms behind the hot-carrier dynamics in 2D perovskite systems upon Rashba band splitting is critical to realizing their applications in new optoelectronic and spintronic devices.

For typical single-layered (001)-oriented 2D hybrid perovskites (*n* = 1) with the general formula A_2_MX_4_ or AMX_4_ (A = organic cation; M = Pb or Sn; X = Cl^−^, Br^−^, or I^−^), the structural, electronic, and photophysical properties are strongly dependent on the nature of the organic spacers (e.g., size, shape, and electron affinity), as well as the configuration of the inorganic layers. Compared to the extensively studied 2D Ruddlesden–Popper (RP) perovskites with monovalent cations, the 2D Dion–Jacobson (DJ) perovskites show shorter interlayer distances and better stability because diammonium cations (+2) can strongly interact with the inorganic layers via both hydrogen bonding and inter-layer van der Waals I···I interactions^21,22^. Herein, we explored and deciphered the hot-carrier cooling processes in two slightly different compositions of 2D DJ perovskites, namely, (3AMP)PbI_4_ and (4AMP)PbI_4_ (3AMP = 3-(aminomethyl)piperidinium; 4AMP = 4-(aminomethyl)piperidinium), by combining ultrafast time-resolved spectroscopy and first-principles calculations. In particular, we seek to understand the effect of Rashba band splitting on the cooling processes. We first confirmed the Rashba band splitting in (4AMP)PbI_4_ by a combination of electronic band structure calculations and temperature-dependent photoluminescence (PL) and time-resolved PL experiments. In both (3AMP)PbI_4_ and (4AMP)PbI_4_, the carrier-hot phonon interactions combined with the hot-phonon effect are responsible for the fast relaxation of hot carriers at early times (less than 1 ps). The nonadiabatic molecular dynamics (NAMD) simulations reveal that the fast intraband relaxations to the band edges are governed by hybrid vibrations and nonadiabatic couplings between the initial and lower states. Moreover, we attribute the fast hot-carrier cooling at longer time delays (up to hundreds of picoseconds) to spin-flip/precession and spin-phonon scattering of hot electrons. In addition, by controlling the spin state of the hot carriers generated upon circular copolarized excitation, we observe slower hot-carrier cooling in (4AMP)PbI_4_ that displays Rashba band splitting. This is also supported by NAMD calculations considering the spin-orbit coupling and decoherence effects.

## Results and Discussion

### Structural, electronic bands, and optical properties of 2D DJ perovskites

**Fig. 1:**
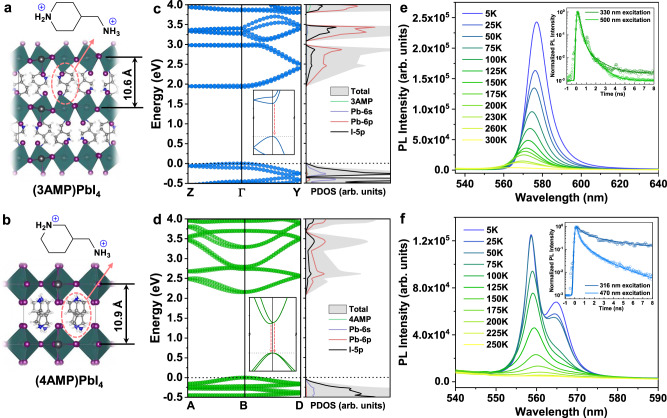
Crystal structure, electronic bands, and PL spectra of 2D Dion–Jacobson perovskites. **a**, **b** Crystal structures of 2D Dion–Jacobson perovskites, (3AMP)PbI_4_ and (4AMP)PbI_4_, together with the chemical structures of the organic cations. **c**, **d** Electronic bands and total and projected density of states (PDOS) of (3AMP)PbI_4_ and (4AMP)PbI_4_ calculated at the DFT HSE06 level with spin-orbit coupling (SOC). Enlarged electronic bands near the valence band maximum and conduction band minimum are shown in the insets. **e**, **f** Temperature-dependent PL spectra of the (3AMP)PbI_4_ and (4AMP)PbI_4_ films, together with the time-resolved PL decays for the emission from the (3AMP)PbI_4_ film at 565 nm and the emission from the (4AMP)PbI_4_ film measured at 560 nm and room temperature. The solid lines represent fits to a biexponential decay function. Note that the observed spikes in (**e**) could be due to the presence of film irregularities that lead to small reflections inside the film.

The 2D Dion–Jacobson perovskite (3AMP)PbI_4_ and (4AMP)PbI_4_ films were prepared from their single crystals using a previously reported method^23,24^ with some modifications (see Materials and Methods for details). The X-ray diffraction (XRD) patterns validate the formation of the 2D phase and the compositional purity (Supplementary Fig. 1a). It also confirms that the perovskite layers are stacked perpendicular to the substrate plane. Atomic force microscopy (AFM) images show a smooth topography of microcrystalline domains that are merged to form compact films (Supplementary Fig. 2). For comparison purposes, 2D Ruddlesden-Popper hybrid perovskites, (PMA)_2_PbI_4_ and (PEA)_2_PbI_4_, were also investigated here. From the crystal structures of (3AMP)PbI_4_ and (4AMP)PbI_4_ (Fig. 1a, b), we find the following: (i) 2D DJ perovskites contain one sheet of divalent +2 cations between the inorganic layers, in which the cations have a balanced positive charge density for both C_5_NH_11_^+^ and NH_3_^+^ (see the electrostatic potential surfaces in Supplementary Fig. 3). (ii) The Pb–I*–*Pb angles in 2D DJ perovskites are much more distorted as they are directly exposed to the organic cations. (iii) The strong hydrogen bonding between the organic cations and the inorganic layers can prevent rotational motion, resulting in more restricted cations or fewer degrees of freedom and short interlayer distances of 10.6 Å and 10.9 Å for (3AMP)PbI_4_ and (4AMP)PbI_4_, respectively. In contrast, the 2D RP perovskites, (PMA)_2_PbI_4_ and (PEA)_2_PbI_4_, contain two sheets of interdigitating monovalent +1 cations with a positive charge density for NH_3_^+^, leading to more flexible layer stacking and larger distances between the inorganic layers: 15.1 Å for (PMA)_2_PbI_4_ and 16.5 Å for (PEA)_2_PbI_4_ (the crystal structures are shown in Supplementary Fig. 4).

The optical band gaps of the (3AMP)PbI_4_ and (4AMP)PbI_4_ films were deduced from the onsets of the absorption spectra to be 2.16 eV and 2.32 eV, respectively (Supplementary Fig. 1b); these values are consistent with the direct band gaps calculated for (3AMP)PbI_4_ at the Γ-point (GGA/PBE: 2.08 eV; HSE + SOC: 1.93 eV) and (4AMP)PbI_4_ at the B-point (GGA/PBE: 2.22 eV; HSE + SOC: 2.16 eV), as shown in Fig. 1c, d. The complete electronic band structures calculated at the GGA/PBE with and without SOC are shown in Supplementary Figs. 5 and 6. Compared to (3AMP)PbI_4_, the better stacking of inorganic layers in (4AMP)PbI_4_ allows stronger interlayer electronic couplings via van der Waals I···I interactions, leading to reduced band gaps via enhanced antibonding interactions and destabilization of the valence bands. The corresponding projected densities of states (PDOSs) suggest that hybridization of Pb-6s and I-5p orbitals mainly contributes to the valence bands, while the Pb-6p orbital has a dominant contribution to the conduction bands.

The (4AMP)PbI_4_ crystal belongs to the P*c* space group and the inorganic layers match perfectly with an averaged equatorial Pb–I–Pb angle of 155°. Such polar octahedral distortions can break the inversion symmetry, leading to Rashba band splitting around the conduction band edge (Fig. 1d). The Rashba splitting coefficient *α*_R_ (defined as 2*E*_R_/*k*_0_, where *k*_0_ is the momentum offset and *E*_R_ is the energy splitting) for (4AMP)PbI_4_ is 1.46 eV·Å, which is on the same order of magnitude as that obtained from recent experiments (*α*_R_ = 2.6 eV·Å)^21^. On the other hand, (3AMP)PbI_4_ belongs to the centrosymmetric P2_1_/*c* space group; here, the inorganic layers stack on top of one another with a larger averaged equatorial Pb–I–Pb angle of 165°, and no band splitting is observed for either the conduction band or valence band edge. Such structural and electronic differences between (3AMP)PbI_4_ and (4AMP)PbI_4_ can be attributed to the stronger hydrogen bonding interactions between H atoms from NH_3_^+^ and I atoms in (4AMP)PbI_4_ (see the comparison of overlap population for hydrogen bonding in Supplementary Table 1). It should be noted that band splitting is also absent in the 2D RP perovskites (PMA)_2_PbI_4_ and (PEA)_2_PbI_4_ due to the absence of tilting of the Pb–I–Pb angles.

To confirm the Rashba band splitting in (4AMP)PbI_4_, we first carried out temperature-dependent PL measurements ranging from 5 K to 300 K. As shown in Fig. 1e, (3AMP)PbI_4_ shows a single emission peak (564.8 nm) at room temperature and exhibits a spectral red-shift for decreasing temperature. In contrast, the emission peak for (4AMP)PbI_4_ starts to split into two for temperatures lower than 150 K (Fig. 1f); and two emission peaks located at 558.7 nm and 564.9 nm are observed at 5 K, which can be attributed to the two split bands (see the insert of Fig. 1f). This also agrees well with recent observations of robust ferroelectricity and Rashba band splitting in (4AMP)PbI_4_ via circularly polarized PL measurements^21^. We further performed time-resolved PL (TRPL) measurements to confirm the band splitting in (4AMP)PbI_4_. For excitation close to the band edge, (3AMP)PbI_4_ shows an average PL lifetime of 0.17 ns, which is slightly shorter than that obtained with high-energy excitation (*τ*_ave_ = 0.27 ns). Similar PL decays are observed in 2D RP perovskites (Supplementary Fig. 7), showing an average PL lifetime of 0.25 ns and 0.34 ns for (PMA)_2_PbI_4_ and (PEA)_2_PbI_4_, respectively. In contrast, (4AMP)PbI_4_ shows a much longer PL lifetime, especially with high-energy excitation (*τ*_ave_ = 7.69 ns). Such slower radiative recombination in (4AMP)PbI_4_ can be attributed to the indirect bandgap nature induced by spin-polarized Rashba band splitting^10,25^.

### Hot carrier cooling processes in 2D DJ perovskites

**Fig. 2:**
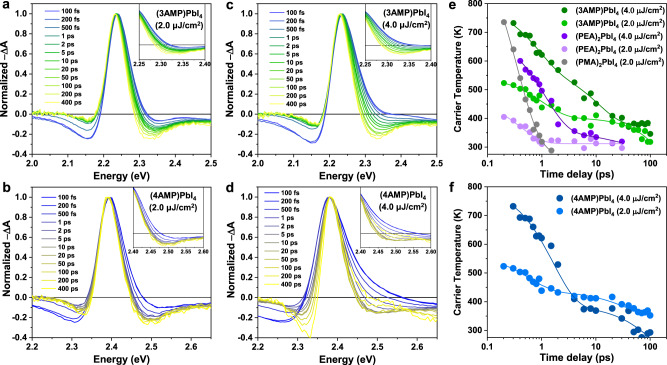
Hot carrier cooling dynamics of 2D Dion–Jacobson perovskites. Normalized transient absorption spectra measured at an excitation wavelength of (**a**, **c**) 330 nm for the (3AMP)PbI_4_ film and (**b, d**) 316 nm for the (4AMP)PbI_4_ film with a pump fluence of 2.0 μJ/cm^2^ and 4.0 μJ/cm^2^. Extracted hot-carrier temperatures as a function of delay time for (**e**) (3AMP)PbI_4_ and (**f**) (4AMP)PbI_4_ at different pump fluences (2.0 μJ/cm^2^ and 4.0 μJ/cm^2^). The hot carrier temperatures of (PMA)_2_PbI_4_ and (PEA)_2_PbI_4_ are shown with gray and purple circles for comparison. The solid lines represent fits to a biexponential decay function.

To assess the hot-carrier cooling properties in 2D DJ perovskite films following excitation at excess energy, we performed femtosecond transient absorption (fs-TA) spectroscopy at different excitation fluences. Figure 2a–d show the normalized TA spectra of 2D DJ perovskite films at high-energy excitation, i.e., 330 nm (3.76 eV) for (3AMP)PbI_4_ and 316 nm (3.92 eV) for (4AMP)PbI_4_, with pump fluences of 2.0 μJ/cm^2^ and 4.0 μJ/cm^2^ to minimize Auger recombination (see Supplementary Note 1). TA spectra at a lower pump fluence of 0.8 μJ/cm^2^ were also obtained and are shown in Supplementary Fig. 8. Note that the carrier densities (*n*_0_) corresponding to pump fluences of 2.0 μJ/cm^2^ and 4.0 μJ/cm^2^ are 8.25 × 10^17^ and 1.65 × 10^18^ cm^−3^ for (3AMP)PbI_4_ and 8.80 × 10^17^ and 1.76 ×10^18^ cm^−3^ for (4AMP)PbI_4_ (see Supplementary Note 2). Upon photoexcitation, the TA spectra of both the (3AMP)PbI_4_ and the (4AMP)PbI_4_ films show photobleaching (PB) peak (−ΔA > 0) with high-energy tails near the bandgap due to band filling effects, as well as broad below-bandgap photoinduced absorption (PIA, −ΔA < 0) and above-bandgap PIA due to the change in the imaginary part of the refractive index^26^. Note that the band gap renormalization is almost negligible in both cases. With time, the high-energy tails gradually narrow, which suggests that the hot-carrier cooling process is accompanied by longitudinal optical (LO) phonon emission^16^.

The hot-carrier temperature (*T*_c_) can be extracted by fitting the high-energy tails of the TA spectra using the Maxwell-Boltzmann function exp[(*E*_f_ – *E*)/*k*_B_*T*_c_], where *k*_B_ is the Boltzmann constant and *E*_f_ is the quasi-Fermi energy (see Supplementary Note 3)^27^. Figure 2e, f show the *T*_c_ evolution following high-energy excitation at different pump fluences. At a low pump fluence of 2.0 μJ/cm^2^, the initial *T*_c_ in the (3AMP)PbI_4_ film is 524 K, and it cools fast with a time constant of 1.25 ps (obtained from the biexponential fittings of initial *T*_c_ decays, see Supplementary Table 3) and an energy loss rate of 1.22 eV/ps (i.e., the initial excess energy divided by the cooling time) until *T*_c_ approaches room temperature. At a higher pump fluence of 4.0 μJ/cm^2^, the initial *T*_c_ of the (3AMP)PbI_4_ film reaches 726 K, and the hot electrons require a longer time to relax to the conduction band edge, giving a time constant of 2.46 ps and an energy loss rate of 0.62 eV/ps. Note that the 2D RP perovskite (PEA)_2_PbI_4_ film shows similar evolutions for the high-energy tails in the TA spectra (Supplementary Fig. 9) but exhibits much lower initial *T*_c_, as well as *T*_c_ time constant (0.36/1.19 ps for (PEA)_2_PbI_4_ at low/high pump fluence), compared to that of (3AMP)PbI_4_. Although the initial *T*_c_ of the (PMA)_2_PbI_4_ film is even higher than that of (3AMP)PbI_4_ under the same pump fluence, *T*_c_ experiences a fast decay with a *T*_c_ time constant of 0.38 ps. It is worth mentioning that the (PMA)_2_PbI_4_ film is not stable and may undergo a phase change under a higher pump fluence of 4.0 μJ/cm^2^, as a new photobleaching signal appears at a wavelength of 408 nm (Supplementary Fig. 10). From these results, it can be concluded that the initial hot-carrier cooling in 2D hybrid perovskites is dominated within the subpicosecond time scale (<1 ps) by the emission of optical phonons, while an additional slow cooling process is observed on a timescale of up to hundreds of picoseconds, especially at a high pump fluence, which can be attributed to the hot-phonon bottleneck effect^15,26^.

The (4AMP)PbI_4_ film also exhibits a high initial *T*_c_ (523 and 723 K) and rapid initial cooling behavior during thermal equilibrium between the LO-phonon population and hot carriers (< 1 ps). The *T*_c_ time constant of (4AMP)PbI_4_ is 1.50 ps at a low pump fluence (energy loss rate of 1.01 eV/ps), and it becomes longer (2.37 ps) at a high pump fluence. The initial fast cooling in (4AMP)PbI_4_ is comparable to that of (3AMP)PbI_4_ and 2D RP perovskites, suggesting similar carrier-phonon interactions in *n* = 1 2D perovskites. Furthermore, compared to (3AMP)PbI_4_, the lower density of states in the conduction bands for (4AMP)PbI_4_ (Fig. 1c, d) will lead to a reduced energy loss rate of hot carriers due to the presence of fewer available relaxation pathways. However, on a timescale of tens to hundreds of picoseconds, the high-energy tails of the photobleaching signals become fast evolving, indicating that an additional channel is involved in accelerating hot carrier cooling. This faster cooling of the hot carriers closer to the band edges, especially at high pump fluence, can be influenced by spin randomizations and flips of hot electrons from the split bands. Thus, we attribute the further fast *T*_c_ decays taking place on the timescale of tens to hundreds of picoseconds to the Rashba band splitting effect, which will be discussed in the following sections.

### Intraband relaxations in 2D DJ perovskites

**Fig. 3:**
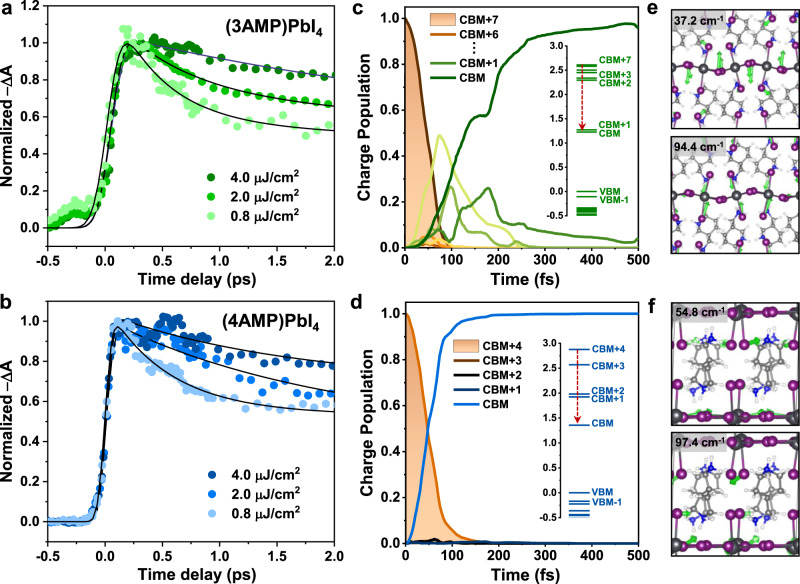
Kinetics and intraband relaxation in 2D Dion–Jacobson perovskites. Normalized transient absorption kinetics (**a**) probed at a wavelength of 554 nm for the (3AMP)PbI_4_ film (excitation at 330 nm) and (**b**) probed at a wavelength of 518 nm for the (4AMP)PbI_4_ film (excitation at 316 nm), with different pump fluences of 0.8 μJ/cm^2^, 2.0 μJ/cm^2^, and 4.0 μJ/cm^2^. The solid lines show best fits to the experimental data with an exponential function. Time evolution of hot electron relaxation (**c**) starting from CBM + 7 for (3AMP)PbI_4_ (excess energy = 1.52 eV) and (**d**) starting from CBM + 4 for (4AMP)PbI_4_ (excess energy = 1.38 eV). Inserts show the electronic energy levels of (3AMP)PbI_4_ and (4AMP)PbI_4_ at the high-symmetry Γ-point and B-point, respectively, involved in the nonadiabatic molecular dynamics calculations (NAMD). Vibrational modes responsible for the intraband relaxation in (**e**) (3AMP)PbI_4_ and (**f**) (4AMP)PbI_4_ (the vibrational motions of the organic cations are omitted). The DFT and NAMD calculations were performed at the GGA/PBE level with SOC.

Figure 3a, b show the normalized TA kinetics of the (3AMP)PbI_4_ and (4AMP)PbI_4_ films probed at the photobleaching peaks with different pump fluences. The buildup of band-edge bleach has been previously used to elucidate hot-carrier cooling processes^28,29^. However, both perovskite films show an instantaneous rise in the photobleaching signals that occur at subpicosecond timescales, i.e., ≤120 fs for (4AMP)PbI_4_ and 120–200 fs for (3AMP)PbI_4_ at pump fluences from 0.8 μJ/cm^2^ to 4.0 μJ/cm^2^. This suggests that (i) the excess energy gained by the electrons in 2D DJ perovskites can simultaneously promote the generation of nonequilibrium LO-phonons and (ii) the Auger heating effect is almost negligible at these pump fluences. The fitting parameters for the TA kinetics for (3AMP)PbI_4_ and (4AMP)PbI_4_ at different pump fluences are given in Supplementary Tables 4 and 5; they show a longer initial decay at high-energy excitations (i.e., *τ*_1_ = 0.45 − 3.50 ps for (3AMP)PbI_4_ and *τ*_1_ = 0.55 − 1.86 ps for (4AMP)PbI_4_) compared to that obtained with low-energy excitations.

To understand the similar rise and initial decays in the TA kinetics as well as the intraband relaxations governed by nonadiabatic coupling among high-energy levels, we performed nonadiabatic molecular dynamics (NAMD) calculations for (3AMP)PbI_4_ and (4AMP)PbI_4_ with consideration of electronic coherence effects. Based on the Kohn–Sham energies for the electronic levels obtained at the GGA/PBE + SOC level (Fig. 3c, d), the NAMD calculations only involved high-energy conduction bands (i.e., electron cooling process) as the effective masses for the electrons are much smaller than those for the holes (i.e., 0.18 *vs*. 0.34 *m*_0_ for (3AMP)PbI_4_; 0.32 *vs*. 0.81 *m*_0_ for (4AMP)PbI_4_). To quantify the intraband relaxations of hot electrons, the population decay curves were fitted using the equation *f*(*t*) = *a*·*exp*(−*t*/*τ*_1_) + (1 − *a*)·*exp*(−(*t*/*τ*_2_)^2^) and the relaxation times were calculated according to τ_intraband_ = *a*·*τ*_1_ + (1 − *a*)·*τ*_2_^30^.

As shown in Fig. 3c, d, the electrons in (3AMP)PbI_4_ undergo a fast relaxation from the initial CBM + 7 (1.52 eV above the conduction band maximum, CBM) to the band edge, and the population of electrons in the initial state rapidly decays to the CBM with a *τ*_intraband_ of 54 fs. For the case of (4AMP)PbI_4_, the population of hot electrons cools down from the initial CBM + 4 to the CBM with a relaxation time of *τ*_intraband_ = 57 fs. The comparable intraband relaxation times agree well with the rise times of the photobleaching signals of (3AMP)PbI_4_ and (4AMP)PbI_4_. The fast intraband relaxation times can be attributed to the direct population transfer from the initial state to the lower states, i.e., to nonvanishing nonadiabatic couplings (NAC; the NAC mappings are shown in Supplementary Fig. 11) among these states, which emerge once spin-orbit coupling is included in the NAMD calculations: NAC[<CBM + 7|CBM + *m*>] (*m* = 0 − 6) = 16 − 28 meV for (3AMP)PbI_4_; NAC[<CBM + 4|CBM + *m*>] (*m* = 0 − 3) = 15 − 27 meV for (4AMP)PbI_4_. These couplings allow the acceleration of hot-carrier cooling via multiple intraband relaxation channels^31^.

Analysis of the experimental Raman spectra, calculated Raman modes, and spectral densities between two conduction bands (i.e., pair states), as shown in Supplementary Fig. 12, confirms that low-frequency Raman modes (at ~40 cm^−1^ for (3AMP)PbI_4_ and ~50 cm^−1^ for (4AMP)PbI_4_) are responsible for the hot-carrier cooling. In a way similar to the vibrational features found in 3D perovskite structures^16,27^, the major low-frequency modes can be assigned to the horizontal Pb−I stretching modes and vertical I–Pb–I rocking modes of the inorganic layer (see the vibrational vectors for the major modes in Fig. 3e, f). Since the organic spacers in 2D perovskites are predominantly coupled to the stretching/rocking vibrations of the inorganic layers, these low-frequency “hybrid phonon” modes can accelerate the hot-carrier cooling processes^13^. Moreover, the hot-carrier cooling in lead halide perovskites is also controlled by electron-phonon coupling (i.e., the interactions between the LO-phonons and electrons)^15,32^, which can be described by the Fröhlich parameter (*α*, see Supplementary Note 4). As given in Supplementary Table 6, 2D DJ perovskites have smaller calculated *α* values (2.53 for (3AMP)PbI_4_ and 2.29 for (4AMP)PbI_4_) than the 2D RP perovskites (3.36 for (PMA)_2_PbI_4_ and 4.29 for (PEA)_2_PbI_4_), indicating that the electronic transitions among the conduction bands in 2D DJ perovskites are less coupled to the vibrations of the inorganic cages. Moreover, (3AMP)PbI_4_ and (4AMP)PbI_4_ have similar screened Coulomb interactions among excited charge carriers, as the dielectric constants for the organic cations are almost the same. Although the lifetimes of the intraband relaxations are slightly longer in 2D RP perovskites because of weaker nonadiabatic couplings (Supplementary Fig. 13), the Coulomb interactions are less screened due to the small dielectric constant of the cations (see Supplementary Note 5), leading to a more significant scattering of the hot carriers with optical phonons.

### Spin-selective hot carrier cooling processes in 2D DJ perovskites

**Fig. 4:**
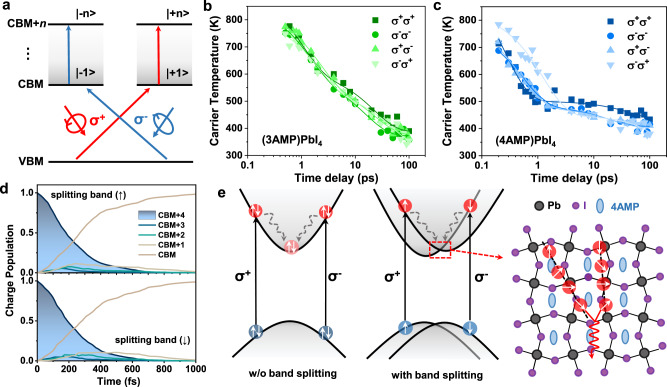
Spin-selective hot carrier relaxation of 2D Dion–Jacobson perovskites. **a** Scheme for the optical selection for the circular polarized TA measurements of 2D DJ perovskite films. Right (left) circular polarized light σ^+^ (σ^−^) couples to the electronic transition from the valence bands to high-level conduction bands. Extracted hot-carrier temperature as a function of delay time of (**b**) (3AMP)PbI_4_ and (**c**) (4AMP)PbI_4_ obtained from cocircular (σ^+^σ^+^ and σ^−^σ^−^) and counter circular (σ^+^σ^−^ and σ^−^σ^+^) polarized pump-probe TA spectra. **d** Time evolution of hot-electron relaxation starting from the two split bands for (3AMP)PbI_4_ calculated with consideration of spin-orbit coupling and decoherence effects. **e** Schematic illustration of the hot-electron relaxation processes in 2D DJ perovskites without and with band splitting and of the spin-flip/precession and scattering processes in (4AMP)PbI_4_.

One way to retard hot-carrier cooling at longer delays in (4AMP)PbI_4_ is to manipulate the hot carriers via band splitting. To confirm and further understand the role of band splitting in the hot-carrier cooling processes, we performed circular polarized transient absorption (CTA) measurements for (3AMP)PbI_4_ and (4AMP)PbI_4_ films. In the CTA measurements, the high-energy excitations were set to be polarized either right-handed circular (σ^+^) or left-handed circular (σ^−^), and a copolarized (counterpolarized) probe pulse was used to probe the hot carrier relaxation from spin-up and spin-down states (see the scheme for the experimental setup in Supplementary Fig. 14). As illustrated in Fig. 4a, spin states with total angular momentum (|+1> or |−1>) and high-energy levels (|+n> or |−n>) are generated using the polarized pump pulse. The normalized cocircular and counter circular pump-probe TA spectra of the (3AMP)PbI_4_ and (4AMP)PbI_4_ films are shown in Supplementary Figs. 15 and 16, and the corresponding fitting parameters for the kinetics probed at the photobleaching peaks are given in Supplementary Table 7.

Figure 4b, c show the *T*_c_ evolution of (3AMP)PbI_4_ and (4AMP)PbI_4_ following high-energy excitation with a pump fluence of 4.0 μJ/cm^2^. For the case of (3AMP)PbI_4_, the *T*_c_ decays are quite similar to those observed in the TA measurements and are not sensitive to the type of circular polarization for the pump and probe. However, (4AMP)PbI_4_ shows a fast initial decay of *T*_c_ and a slower decay of tens of picoseconds when *T*_c_ approaches 400 K, especially with the copolarized σ^+^σ^+^ pump and probe. The extracted *T*_c_ lifetimes and the initial decays (τ_1_) for the (4AMP)PbI_4_ film are 4–6 ps and 2–3.5 ps, respectively, both of which are much higher than those obtained by the TA measurements with the same pump fluence. Therefore, the Rashba band splitting in (4AMP)PbI_4_ can induce an additional barrier to spin flipping because |+n> and |−n> are present in different momentum valleys, as well as reduced spin-phonon scattering of hot electrons from two different spin states. We further performed NAMD calculations by treating the hot electrons from two split conduction bands with consideration of electronic decoherence effects as the high-energy levels of (4AMP)PbI_4_ are well separated^33^. As shown in Fig. 4d, the lifetimes of hot electrons in (4AMP)PbI_4_ with spin-up and spin-down states are ~228 fs, which is much longer than that obtained with consideration of coherence effects and agrees well with the rise time (*τ*_rise_ = ~190–210 fs) obtained from the CTA kinetics. However, (3AMP)PbI_4_ retains a similar rise time (*τ*_rise_ = ~180–200 fs) as compared to that obtained from TA kinetics following the same pump fluence (4 μJ/cm^2^).

The schematic illustration in Fig. 4e depicts the hot-carrier cooling processes in 2D DJ and RP perovskites. In all cases, the initial cooling processes are mainly governed by the nonequilibrium LO-phonon population (i.e., the scattering of hot electrons with LO-phonons). Then, the hot phonon emission contributes to slow hot-carrier cooling up to tens of picoseconds, especially at a high pump fluence. Moreover, the hot-carrier cooling process can be retarded by Coulomb screening effects, as the macroscopic electric field induced by the out-of-phase displacements of the atoms is weakened by Coulomb screening. For (4AMP)PbI_4_, the Rashba band splitting means that the hot-carrier cooling is not only dependent on the interactions between hot electrons and LO-phonons but also affected by spin-dependent scattering of hot electrons^34^. The D’yakonov–Perel’ (DP) mechanism dominates in (4AMP)PbI_4_ in which there is no inversion symmetry, and the Rashba effect induces spin precession randomized by scattering due to the SOC-induced internal effective magnetic field^35^. In addition, strong spin-phonon scattering can decrease the hot-carrier lifetime, as it gives rise to faster spin randomization and flip. To minimize spin-dependent scattering, hot carriers with spin-up or spin-down states can be manipulated through circular copolarized light excitation; in that instance, the hot-carrier temperature approaches 400 K and has a longer lifetime.

In summary, we have demonstrated the significant influence of Rashba band splitting on the hot-carrier cooling dynamics in 2D Dion–Jacobson hybrid perovskites. Electronic band structure calculations and measurements of the temperature-dependent PL spectra and PL decay were used to verify the presence of Rashba band splitting in (4AMP)PbI_4_, which originates from the symmetry breaking by in-plane equatorial distortions. The scattering between hot electrons and LO-phonons governs the initial fast cooling in both (3AMP)PbI_4_ and (4AMP)PbI_4_. The spin-dependent scattering of hot electrons generated by the Rashba effect can accelerate hot-carrier cooling in (4AMP)PbI_4_. Importantly, the cooling process can be slowed down by controlling the spin states of the hot carriers with circular copolarized excitation. Our findings highlight the importance of Rashba band splitting in two-dimensional perovskites as a powerful tool to control hot-carrier cooling dynamics, which can be exploited to design and synthesize 2D materials with promising optoelectronic and spintronic applications.

## Methods

### Materials

Lead(II) oxide (PbO > 99%), lead(II) iodide (PbI_2_ > 99%), 3-(aminomethyl)piperidine (3AMP), 4-(aminomethyl)piperidine (4AMP), benzylamine, hypophosphorous acid (H_3_PO_2_), N,N’-dimethylformamide (DMF), dimethyl sulfoxide (DMSO) and diethyl ether (DEE) were purchased from Sigma Aldrich and used without further purification. Phenylethylammonium iodide (PEAI) was purchased from GreatCell Solar, and hydroiodic acid (HI, 55–58%) was obtained from Alfa Aesar.

### Synthesis of 2D perovskite single crystals

For the (3AMP)PbI_4_ and (4AMP)PbI_4_ single crystals, 1 mmol of PbO (223.2 mg) was dissolved in 4 mL of HI solution under boiling conditions for 5 min. In a separate vial, 1 mmol of 3AMP (114.0 mg) was dissolved in 0.5 mL of H_3_PO_2_. Then, under boiling conditions, H_3_PO_2_ neutralized 3AMP solution was added to the PbO-containing vial. The solution was allowed to boil for another 5 min and then slowly cooled to room temperature at a cooling rate of 3 °C/hour. The (4AMP)PbI_4_ crystals were prepared using a similar method. For the (PMA)_2_PbI_4_ and (PEA)_2_PbI_4_ single crystals, PbI_2_ was used as the Pb precursor, and the synthesis methods have been reported in our previous studies^24,36^.

### Fabrication of 2D perovskite thin films

The solutions of single crystals were prepared in 1 mL of mixed solvent (DMF:DMSO = 80:20) to reach a concentration of 100 mg/mL. To fabricate the thin films, 100 μL of single crystal solution was pipetted onto the glass substrate and spin-coated at 2000 RPM for 60 sec. After 40 sec, 700 μL of DEE was added as an antisolvent. Then, the (3AMP)PbI_4_ and (4AMP)PbI_4_ films were annealed at 140 °C for 5 min, and the (PMA)_2_PbI_4_ and (PEA)_2_PbI_4_ films were annealed at 80 °C for 5 min. The thicknesses of 2D DJ perovskite films were ~300 nm as measured via the thickness profiler; the root mean square roughness (σ_RMS_) was 2.6 nm for (3AMP)PbI_4_ and 8.7 nm for (4AMP)PbI_4_ (see Supplementary Fig. 2).

### X-ray diffraction measurements

X-ray diffraction patterns of the (3AMP)PbI_4_ and (4AMP)PbI_4_ films were recorded using a Bruker D8 Advance (40 kV, 40 mA) with CuK_α1_ radiation (λ = 1.5406 Å) operating at a step size of 0.02° and a speed of 0.4 sec/step at room temperature.

### Steady-state absorption measurements

Absorption spectra of the (3AMP)PbI_4,_ (4AMP)PbI_4,_ (PMA)_2_PbI_4_ and (PEA)_2_PbI_4_ films were recorded in absorbance mode using a LAMBDA 1050 (Perkin Elmer).

### Raman measurements

Raman measurements were performed using a Witec Apyron instrument equipped with a 1064-nm laser and a Zeiss EC Epiplan–Neofluar 100×/0.9 NA Objective lens. The signal integration time was set at 45 s.

### Temperature-dependent PL measurements

Temperature-dependent PL measurements were carried out for the (3AMP)PbI_4_ and (4AMP)PbI_4_ films by using a 488 nm Ar+ laser. The signals were detected by Andor Shamrock spectrograph attached to Andor Newton CCD camera. Cryogenic measurements were carried out in a closed-cycle cryostat under vacuum with temperatures ranging between 5 K and 300 K.

### Time-resolved PL measurements

Time-resolved PL measurements were performed using the time-correlated single-photon counting technique (TCSPC). The different excitation wavelengths used for the 2D perovskite films were tuned using a parametric optical amplifier (Newport, Spectra Physics) that was pumped with an ultrafast Ti:sapphire amplifier (800 nm, 100 fs, 1 kHz, Astrella, Coherent). The energy at each excitation wavelength was modulated by a pair of variable neutral density filters (Thorlabs). The excitation beam entered a Halcyone setup (Ultrafast Systems) where it was focused onto the sample, and the resulting photoluminescence was collected and recollimated by using a pair of parabolic mirrors, passed through longpass filters (450 nm, 550 nm, Newport), and finally focused onto an optical fiber directed towards the monochromator and detector. To ensure that less than 1% of the excitation events resulted in photon detection, an additional variable ND filter was used before the optical fiber. The 2D perovskite films were measured in the reflection arrangement of the system, and the obtained TCSPC histograms were fitted using the Lavenberg–Marquart algorithm implemented in Ultrafast Systems software. The overall setup has a time resolution of 110 ps. The detection fiber optics are coupled to a multichannel spectrometer with a CMOS sensor that has a 1.5-nm intrinsic resolution with maximum spectral acquisition rate of 9500 spectra/s.

### Femtosecond transient absorption measurements

Femtosecond transient absorption measurements were performed for the 2D perovskite films by using a multipass amplified Ti:sapphire laser (800 nm laser pulses with a 7 mJ/pulse; pulse width of ~100 fs with a repetition rate of 1 kHz, Astrella from Coherent) in conjunction with Helios spectrometers. Different excitation pump pulses (see the summary in Supplementary Table 2) were generated after passing through a fraction of the 800 nm beam into a spectrally tunable (240–2600 nm) optical parametric amplifier (Newport Spectra-Physics). The probe pulses (UV-Visible and NIR wavelength continuum, white light) were generated by passing another fraction of the 800 nm pulses through a 2 mm thick calcium fluoride (CaF_2_) crystal. The absorption change (ΔA) was measured with respect to the time delay and wavelength. Circular polarized TA measurements were performed using the same setup with an adjustment (see Supplementary Fig. 14). A half waveplate (λ/2) and a fixed circular polarizer were set to control the pump polarization. The probe pulses were detected either parallel or perpendicular to the pump pulses, which was controlled by a variable polarizer.

### Density functional theory (DFT) calculations

DFT calculations were carried out for 2D perovskites, (PMA)_2_PbI_4_, (PEA)_2_PbI_4_, (3AMP)PbI_4_, and (4AMP)PbI_4_, using the projector-augmented wave (PAW) method as implemented in the VASP code^37,38^. The generalized gradient approximation (GGA) with the Perdew–Burke–Ernzerhof (PBE) exchange-correlation functional was used. A uniform k-mesh grid of 4 × 4 × 2 was used for (PMA)_2_PbI_4_, (PEA)_2_PbI_4_, and (4AMP)PbI_4_, and 4 × 2 × 2 was used for (3AMP)PbI_4_. The plane-wave basis set cutoffs for the wavefunctions were set at 450 eV. Starting from the experimental lattice parameters (CCDC 1831521 for (3AMP)PbI_4_ and CCDC 1831525 for (4AMP)PbI_4_^22^), the atomic positions for all 2D perovskites were fully relaxed by relaxing both the cell parameters and the atomic positions until the supercells had forces of less than 0.01 eV/Å on each atom. The Heyd–Scuseria–Ernzerhof hybrid functional (HSE06), including spin-orbit coupling, was used to calculate the electronic band structures (Fig. 1c, d).

The Raman and far-infrared vibrational mode positions and intensities of the 2D perovskites were calculated by using the Phonon code as implemented in the Quantum ESPRESSO (QE) package^39,40^. The local density approximation (LDA) exchange-correlation functional with norm-conserving pseudopotentials was used, and the plane-wave basis set cutoffs for the wavefunctions were set at 90 Ry with a self-consistency threshold of 10^−14^ Ry. The SOC was not included in the Raman calculations, as it plays a less significant role than the geometry in the description of the vibrational properties of heavy metal-based perovskite systems.

### Nonadiabatic molecular dynamics (NAMD) calculations

The crystal structures of the 2D perovskites were further optimized at the GGA/PBE level using the PWSCF code as implemented in the QE package^39^. Ultrasoft pseudopotentials were used considering spin-orbit coupling. Uniform Brillouin zone grids of 4 × 4 × 2 k-mesh for (PMA)_2_PbI_4_, (PEA)_2_PbI_4_, and (4AMP)PbI_4_ and 4 × 2 × 2 for (3AMP)PbI_4_ were employed, and the plane-wave basis set cutoffs for the wavefunctions and charge density were set at 40 Ry and 300 Ry, respectively. The optimized crystal structures of the 2D perovskites were considered as the starting point for calculating the ground-state molecular dynamics trajectories, where a 1000 fs trajectory for the system was obtained. The Andersen thermostat was used to control the temperature of the system at 300 K. The initial 1000 fs for the trajectories with a 1 fs time step were obtained for nuclear thermalization, and the subsequent 2000 fs were used for the NAMD calculations.

NAMD calculations for the 2D perovskites were carried out using the PYXAID2 code^33,41^. The fewest-switches surface hopping algorithm^42^ implemented within time-dependent DFT was used to investigate the hybrid perovskite systems^43–45^. Starting from the time-dependent Schrödinger equation $i\hslash \frac{\partial {\varPsi }_{n}\left({\bf{r}},t\right)}{\partial t}=H\left({\bf{r}},{\bf{R}},{t}\right){\Psi }_{n}\,\left({\bf{r}}{\boldsymbol{,}}{\rm{t}}\right)$ and Kohn–Sham orbitals ${\Psi }_{n}\,\left({\bf{r}}{\boldsymbol{,}}{\rm{t}}\right){\boldsymbol{=}}\mathop{\sum}\limits_{k}{C}_{k}^{n}\left(t\right){\Phi }_{k}\left({\bf{r}}{\rm{;}}{\bf{R}}\right)$, the hot carrier relaxations were investigated by calculating the average energy and population of charge carriers from several excited states. The probability of transition between adiabatic states *i* and *j* can be calculated using the wavefunction expansion coefficients and coupling, defined as ${d}_{ij}=-i\hslash \langle {\Phi }_{i}|\frac{\partial }{\partial t}{|\Phi }_{j}\rangle$. Detailed descriptions for the NAMD theory can be found in Refs. ^33,41^. A total of 1500 geometries were randomly selected from the adiabatic trajectories and used as initial conditions in the NAMD calculations.

## Supplementary information

Supplementary Information

## Data Availability

The data that support the findings of this study are available from the corresponding author upon reasonable request.

## Code availability

Vienna ab initio simulation package (VASP) for the periodic DFT calculations is available at https://www.vasp.at/. Quantum Espresso code for the phonon calculations is freely available at https://www.quantum-espresso.org/. PYXAID code for the nonadiabatic molecular dynamics calculations is available at https://github.com/quantum-dynamics-hub/pyxaid2.
