# Supplementary material for: Manipulation of hot carrier cooling dynamics in two-dimensional Dion–Jacobson hybrid perovskites via Rashba band splitting
Source: Nat Commun. 2021 Jun 28;12:3995. doi: 10.1038/s41467-021-24258-7 (PMC8239041; doi:10.1038/s41467-021-24258-7)
Supplement: Supplementary file 1 — Supplementary Information [file 41467_2021_24258_MOESM1_ESM.pdf]

Supplementary Information

Manipulation of Hot Carrier Cooling Dynamics in Two-dimensional Dion-Jacobson Hybrid Perovskites via Rashba Band Splitting

Jun Yin,[1,2,¶] Rounak Naphade,[2,¶] Partha Maity,[1,2,¶] Luis Gutiérrez-Arzaluz,[1,2] Dhaifallah Almalawi,[3] Iman S. Roqan,[3] Jean-Luc Brédas,[4,*] Osman M. Bakr,[2,*] Omar F. Mohammed[1,2,*]

[1] Advanced Membranes and Porous Materials Center, Division of Physical Science and Engineering, King Abdullah University of Science and Technology, Thuwal 23955-6900, Kingdom of Saudi Arabia

[2] KAUST Catalysis Center, Division of Physical Sciences and Engineering, King Abdullah University of Science and Technology, Thuwal 23955-6900, Kingdom of Saudi Arabia

[3] Division of Physical Sciences and Engineering, King Abdullah University of Science and Technology, Thuwal 23955-6900, Kingdom of Saudi Arabia

[4] Department of Chemistry and Biochemistry, The University of Arizona, Tucson, Arizona 85721-0088, United States

* Corresponding authors. Email: omar.abdelsaboor@kaust.edu.sa (O.F.M.); osman.bakr@kaust.edu.sa (O.M.B.); jlbredas@arizona.edu (J.L.B)

¶ Contributed equally to this work

Supplementary Note 1: Minimizing the Auger heating effect

Hot-carrier re-excitation via interband Auger recombination of carriers at band edges (also called Auger heating) can induce slow hot-carrier cooling in the tens to hundreds of picosecond time range, particularly at high pump fluence.[1] In our work, we kept the pump fluence low, in the range of 0.8−4.0 μJ/cm^2, in order to minimize the Auger heating effect.

Supplementary Note 2: Carrier density calculations

The carrier density is estimated via $n_0 = \frac{P\alpha}{\hbar\omega}$, where P is the pump fluence, α is the absorption coefficient of the film, and $\hbar\omega$ is the photon energy. The estimated carrier densities for $(3AMP)PbI_4$ and $(4AMP)PbI_4$ are as follows:

**Compounds**	**Photon Energy (eV)**	**Absorption Coefficient (cm^{-1})**	**Pump Fluence (μJ/cm^2)**	**Carrier Density (cm^{-3})**
$(3AMP)PbI_4$	3.76	2.49×10^5	0.8 to 4.0	3.31×10^{17} to 1.65×10^{18}
$(4AMP)PbI_4$	3.92	2.77×10^5	0.8 to 4.0	3.53×10^{17} to 1.76×10^{18}

Supplementary Note 3: Hot-carrier temperatures (*T*c)

T_c values were extracted by fitting the high-energy tails of the TA spectra using the Maxwell-Boltzmann function. When the difference between the carrier energy and the Fermi level is larger than k_BT (*i.e.*, $E-E_f > k_B$T), the Fermi-Dirac distribution can be approximately described by the Maxwell-Boltzmann distribution: $\frac{1}{1+exp\frac{E-E_f}{k_BT}} \approx exp\frac{E-E_f}{k_BT}$, which is a generally accepted practice to extract the T_c value. More details can be found in refs. [1, 2, 3].

Supplementary Note 4: Electron-phonon coupling calculations

The electron-phonon coupling is described by the dimensionless Fröhlich parameter α, defined as: $\alpha = \frac{1}{4\pi\varepsilon_0}\frac{e^2}{\hbar}\left(\frac{1}{\varepsilon_\infty} - \frac{1}{\varepsilon_s}\right)\sqrt{\frac{m}{2\hbar\omega}}$, where ε_0 is the dielectric permittivity of vacuum; ε_∞ and ε_s are the optical and static dielectric constants, respectively; e is the charge of the carrier; $2\pi\hbar$ is Planck's constant; m is the bare electron effective mass; and ω is the characteristic angular frequency of the longitudinal optical (LO) phonon mode. ε_s was calculated using the linear response method as implemented in the Quantum Espresso package.[4] The dielectric function at far-infrared frequencies was obtained using the following equation: $\varepsilon(\omega) = 1 + \sum_{n=1,Nph}\frac{F_n^2}{\omega_n^2-(\omega+i\eta)^2} + \frac{F_{el}^2}{\omega_{el}^2-(\omega+i\eta_{el})^2}$, where n is the index of the N_{ph} phonon modes; ω_n is the n_{th} phonon frequency; ω_{el} is the electronic band gap; F_n and F_{el} are the oscillator strengths; and η and η_{el} are the broadening factors. The details of the calculations of the far-infrared dielectric function can be found in refs [5, 6].

Supplementary Note 5. Dielectric constant of organic cation layers

The dielectric constant (ε) of PEA is 3.3.[7] Although the experimental dielectric constants of 3AMP and 4AMP are not available, their values can be expected to be higher than that of PEA as the additional functional group ($-CH_2-NH_3^+$) further increases the dielectric constant of piperidine (ε = 5.9).

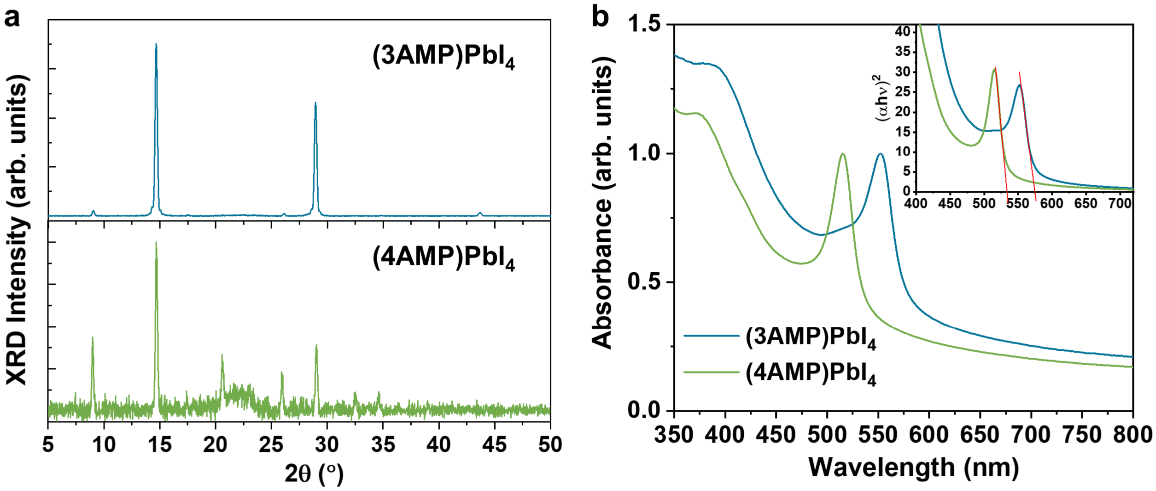

Supplementary Figure 1. Optical characterizations of 2D DJ perovskites. (**a**) X-ray diffraction (XRD) patterns and (**b**) absorption spectra for (3AMP)PbI_4 and (4AMP)PbI_4 films (the Tauc plots of the absorption spectra are shown in the insets).

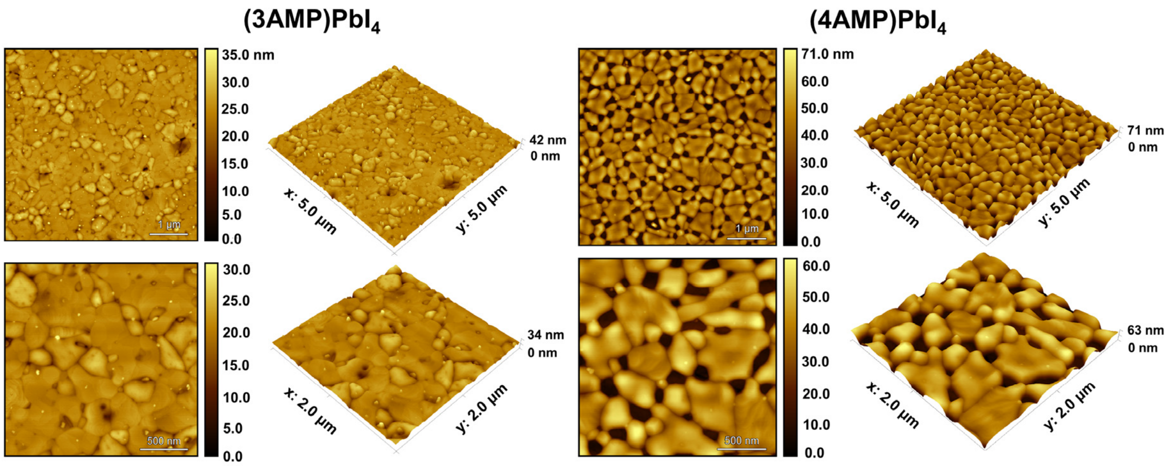

Supplementary Figure 2. Atomic force microscopy (AFM) images of (3AMP)PbI_4 and (4AMP)PbI_4 films.

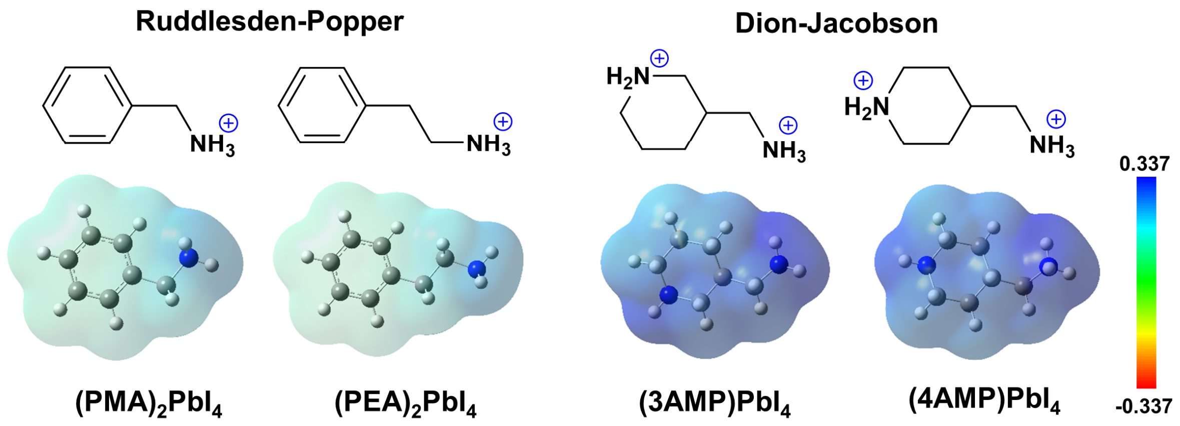

Supplementary Figure 3. Molecular structures and electrostatic potential surfaces of organic spacers phenmethylammonium (PMA) and phenethylammonium (PEA) in the 2D Ruddlesden-Popper perovskites, and 3-(aminomethyl)piperidinium (3AMP) and 4-(aminomethyl)piperidinium (4AMP) in the 2D Dion-Jacobson perovskites.

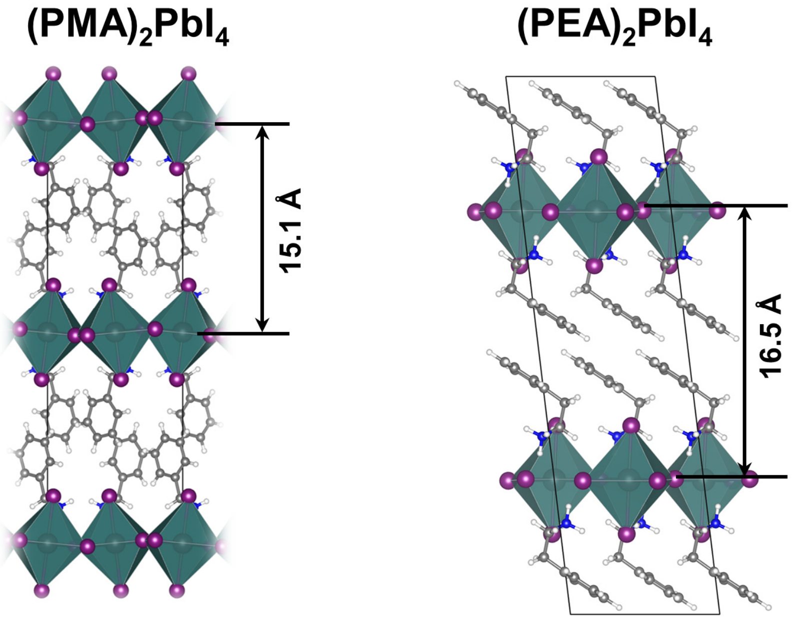

Supplementary Figure 4. Crystal structures for 2D RP perovskites, $(PMA)_2PbI_4$ and $(PEA)_2PbI_4$, together with the chemical structures of organic cations and the distances between inorganic layers.

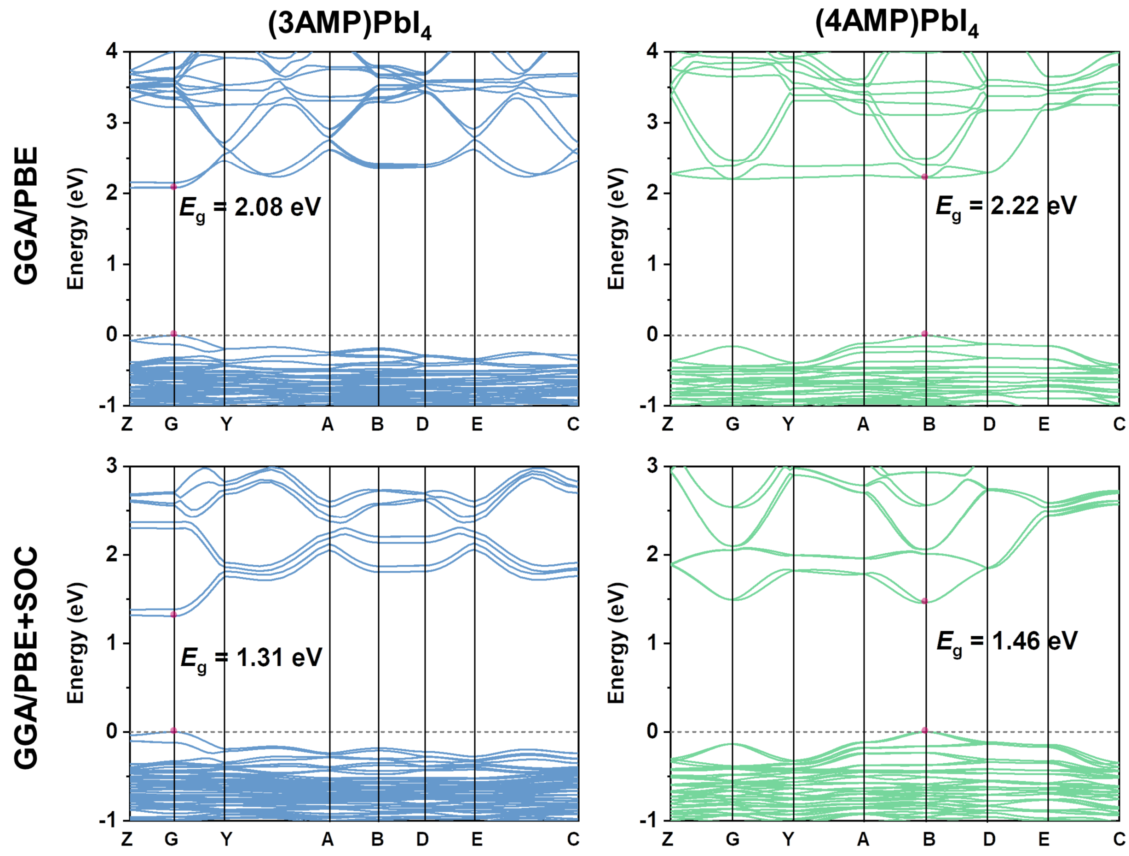

Supplementary Figure 5. Electronic band structures for 2D DJ perovskites $(3AMP)_2PbI_4$ and $(4AMP)_2PbI_4$, calculated at the GGA/PBE level of theory without and with spin-orbit coupling (SOC).

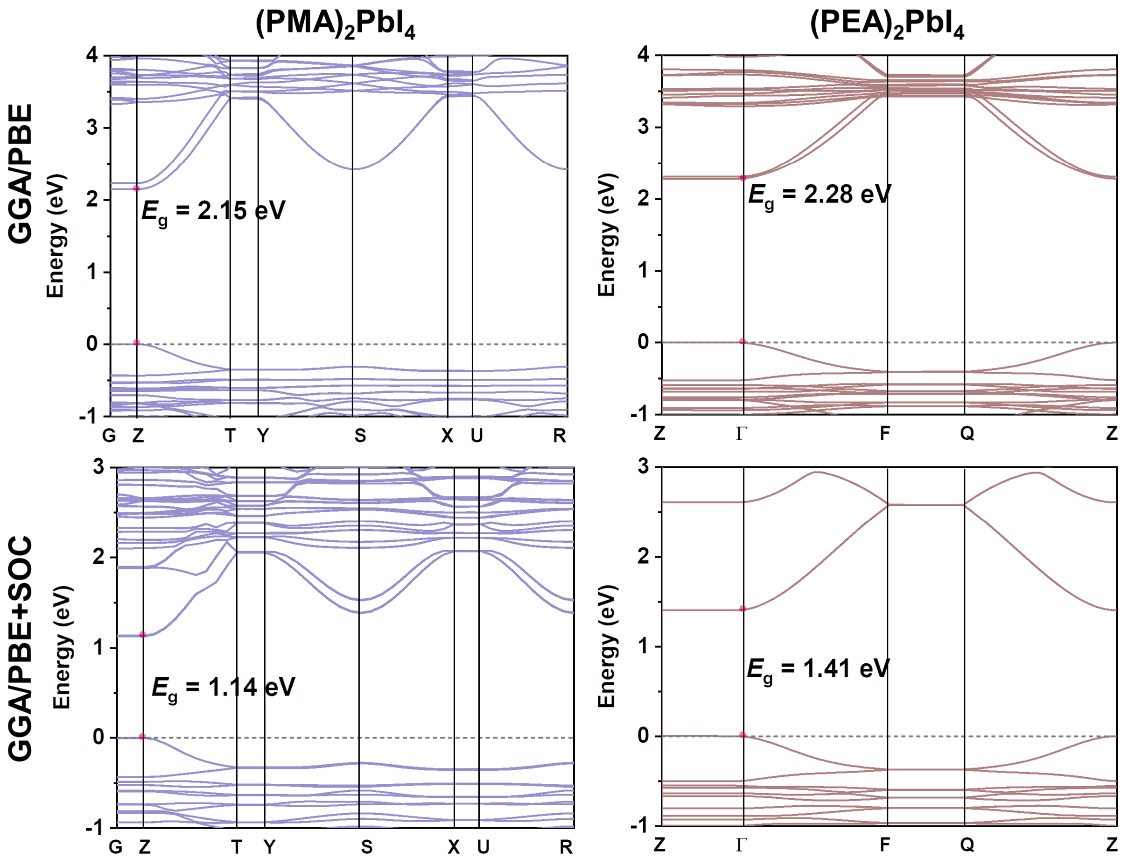

Supplementary Figure 6. Electronic band structures for 2D RP perovskites $(PMA)_2PbI_4$ and $(PEA)_2PbI_4$, calculated at the GGA/PBE+SOC level of theory without and with spin-orbit coupling (SOC).

Supplementary Table 1. Hydrogen bond lengths and overlap population for the hydrogen bonding in $(3AMP)PbI_4$ and $(4AMP)PbI_4$.

H-bonding of $(3AMP)PbI_4$	Bond Length (Å)	Overlap Population (ρ, a.u.)	Total ρ (a.u.)
N-H_2···I	3.30	0.0236	0.1537
	3.17	0.0341	
	2.60	0.0960	
N-H_3···I	3.25	0.0276	0.1389
	2.80	0.0686	
	3.42	0.0181	
	3.38	0.0246	

H-bonding of $(4AMP)PbI_4$	Bond Length (Å)	Overlap Population (ρ, a.u.)	Total ρ (a.u.)
N-H_2···I	3.03	0.0386	0.1521
	3.25	0.0288	
	3.19	0.0327	
	2.89	0.0521	
N-H_3···I	3.05	0.0423	0.1743
	2.73	0.0797	
	3.27	0.0255	
	3.19	0.0268	

Supplementary Table 2. Excitation wavelengths (energies) used for the TA, CTA, and time-resolved PL measurements on $(3AMP)PbI_4$, $(4AMP)PbI_4$, $(PMA)_2PbI_4$, and $(PEA)_2PbI_4$ films.

Film	GSB Position	High-Energy Excitation	Low-Energy Excitation
$(3AMP)PbI_4$	554 nm (2.24 eV)	330 nm (3.76 eV)	500 nm (2.48 eV)
$(4AMP)PbI_4$	517 nm (2.40 eV)	316 nm (3.92 eV)	470 nm (2.64 eV)
$(PMA)_2PbI_4$	520 nm (2.39 eV)	318 nm (3.90 eV)	472 nm (2.63 eV)
$(PEA)_2PbI_4$	519 nm (2.39 eV)	318 nm (3.90 eV)	472 nm (2.63 eV)

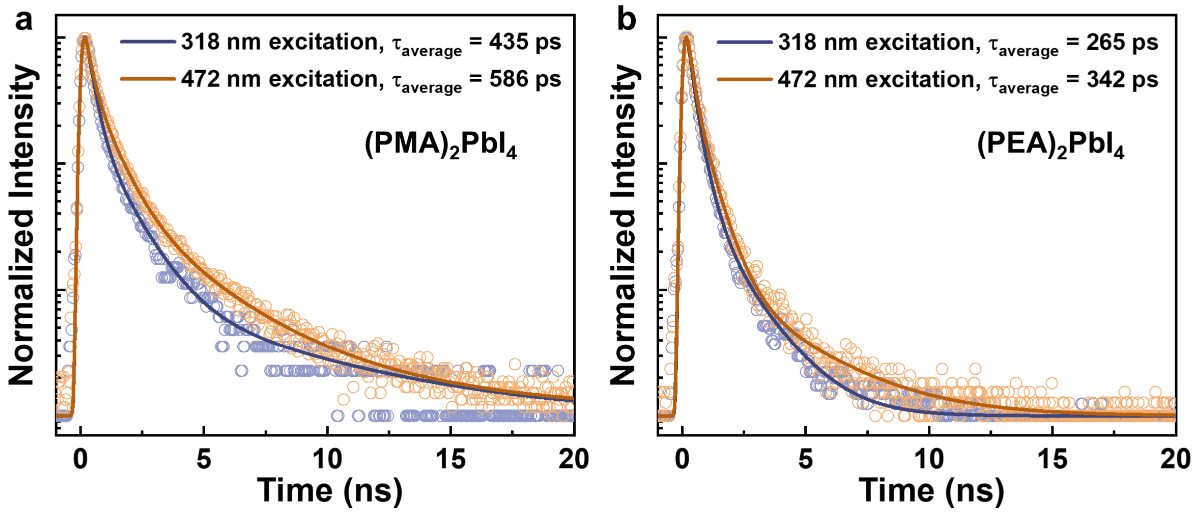

Supplementary Figure 7. Time-resolved PL decays for (**a**) the emission at 565 nm of the $(PMA)_2PbI_4$ film and (**b**) the emission at 560 nm of the $(PEA)_2PbI_4$ film at excitation wavelengths of 318 and 472 nm.

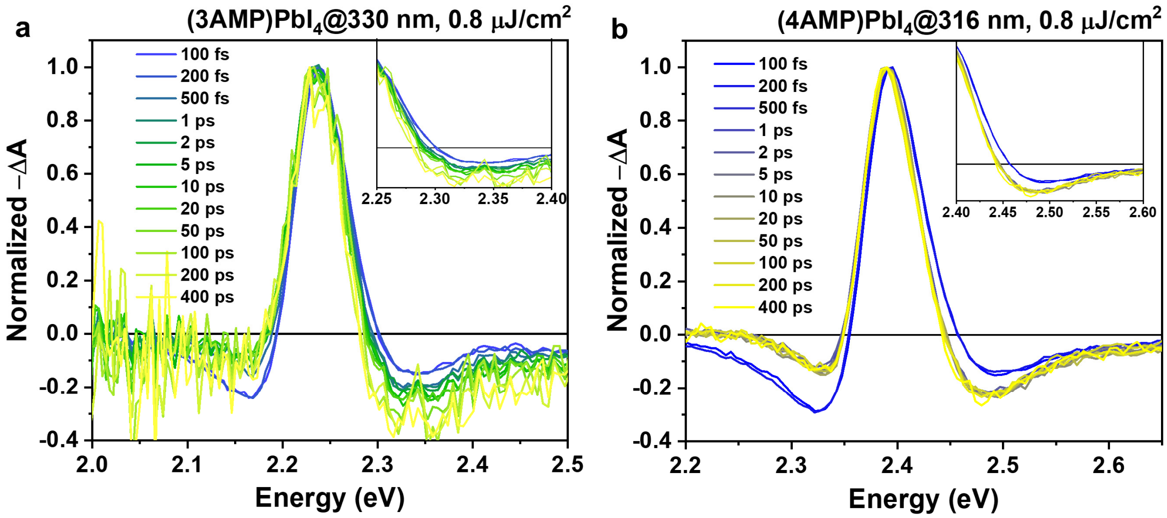

Supplementary Figure 8. Normalized transient absorption spectra at the excitation wavelength of (**a**) 330 nm for the (3AMP)PbI_4 film and (**b**) 316 nm for the (4AMP)PbI_4 film with pump fluence of 0.8 μJ/cm^2.

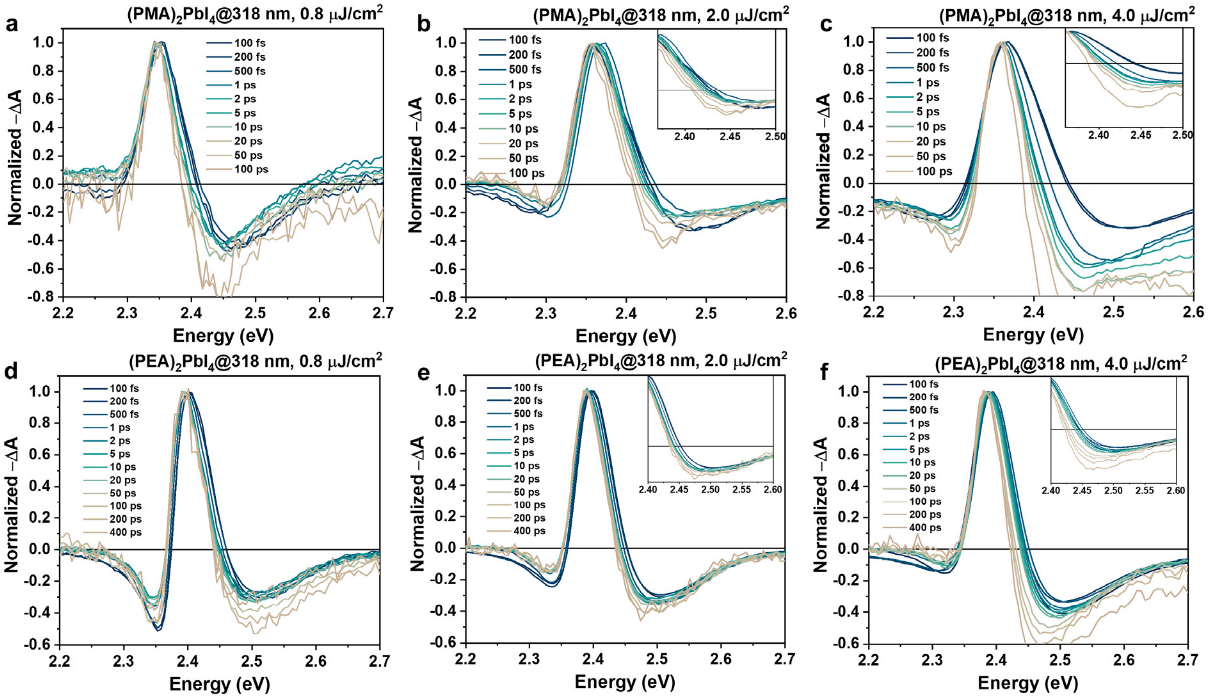

Supplementary Figure 9. Normalized transient absorption spectra at the excitation wavelength of 318 nm for (**a, b, c**) the $(PMA)_2PbI_4$ film and (**d, e, f**) the $(PEA)_2PbI_4$ film, with pump fluences of 0.8, 2.0, and 4.0 μJ/cm^2, respectively.

Supplementary Table 3. Fitting parameters of hot-carrier cooling temperature (T_c) decays in $(3AMP)PbI_4$ and $(4AMP)PbI_4$. The time constants for T_c were obtained via biexponential fittings of the initial T_c decay using the equation: $\tau = a_1 \cdot \tau_1 + a_2 \cdot \tau_2$.

Film	Pump Fluence ($\mu J/cm^2$)	a_1	τ_1 (ps)	a_2	τ_2 (ps)
$(3AMP)PbI_4$	2.0	41%	0.43	59%	1.81
	4.0	48%	0.48	52%	4.27
$(4AMP)PbI_4$	2.0	83%	0.66	17%	5.55
	4.0	85%	1.35	15%	8.31

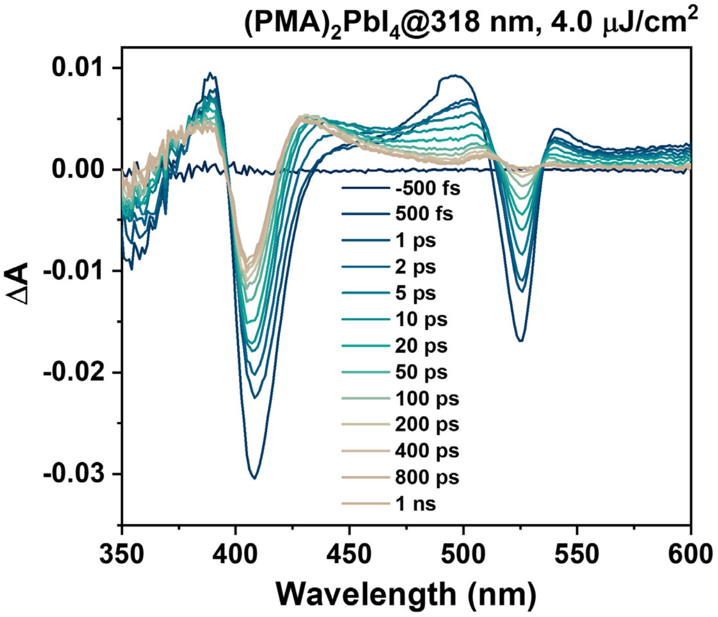

Supplementary Figure 10. Transient absorption spectra at the excitation wavelength of 318 nm for the $(PMA)_2PbI_4$ film with pump fluence of 4.0 μJ/cm^2. A new photobleaching signal appears at 408 nm due to a phase change.

Supplementary Table 4. Fitting parameters of the transient absorption kinetics probed at 554 nm for the $(3AMP)PbI_4$ film (excitation wavelength of 330 nm) and at 518 nm for the $(4AMP)PbI_4$ film (excitation wavelength of 316 nm) with different pump fluences.

Film	Pump Fluence (μJ/cm²)	A_1	τ_1 (ps)	A_2	τ_2 (ps)	A_3	τ_3 (ps)
$(3AMP)PbI_4$	0.8	53%	0.45	32%	15	15%	720
	2.0	43%	1.00	33%	28	24%	373
	4.0	56%	3.50	29%	60	15%	662
$(4AMP)PbI_4$	0.8	48%	0.55	26%	72	26%	968
	2.0	33%	1.53	42%	63	25%	658
	4.0	52%	1.86	33%	49	15%	334

Supplementary Table 5. Fitting parameters of the transient absorption kinetics probed at 554 nm for the $(3AMP)PbI_4$ film (excitation wavelength of 500 nm) and at 518 nm for the $(4AMP)PbI_4$ film (excitation wavelength of 470 nm) with different pump fluences.

Film	Pump Fluence (μJ/cm²)	A_1	τ_1 (ps)	A_2	τ_2 (ps)	A_3	τ_3 (ps)
$(3AMP)PbI_4$	0.8	44%	0.1	24%	45	22%	525
	2.0	16%	0.2	46%	4.8	38%	176
	4.0	33%	0.4	38%	8.0	29%	232
$(4AMP)PbI_4$	0.8	17%	0.2	54%	37	29%	666
	2.0	20%	0.4	51%	32	29%	473
	4.0	49%	1.1	37%	61	14%	670

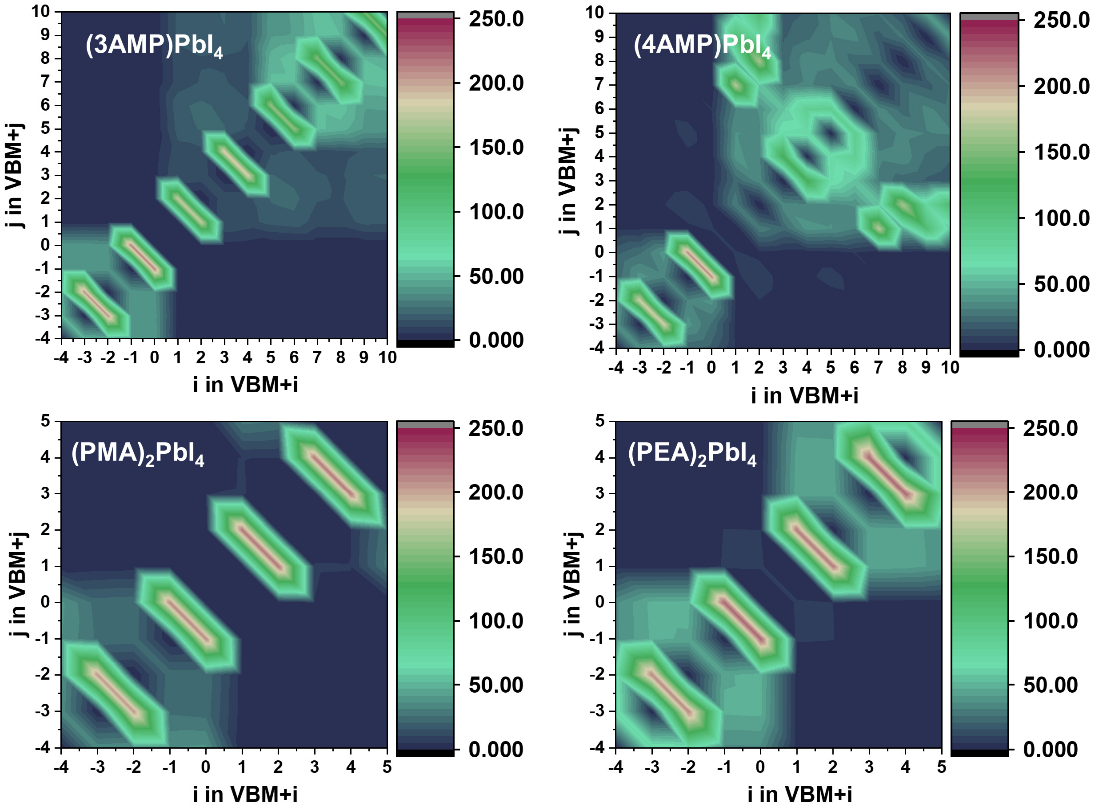

Supplementary Figure 11. Average nonadiabatic couplings (NACs, in meV) between electronic states for $(3AMP)PbI_4$, $(4AMP)PbI_4$, $(PMA)_2PbI_4$, and $(PEA)_2PbI_4$, as obtained from nonadiabatic molecular dynamics (NAMD) simulations.

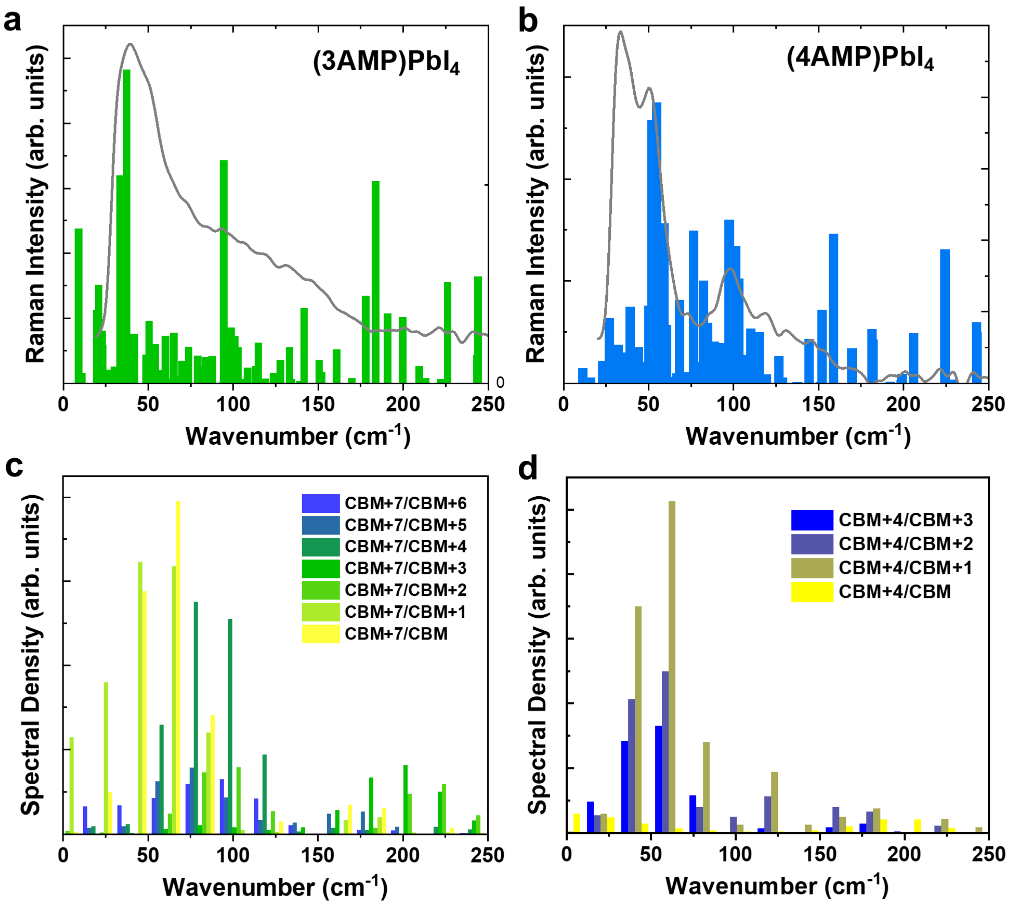

Supplementary Figure 12. Low-frequency vibrational modes. (**a, b**) Experimental Raman spectra (grey lines) and calculated Raman peaks and intensities for $(3AMP)PbI_4$ and $(4AMP)PbI_4$ obtained at LDA level of theory. (**c, d**) Spectral densities obtained by calculating the Fourier transforms of the autocorrelation functions for the fluctuations of the energy differences between two conduction bands in $(3AMP)PbI_4$ and $(4AMP)PbI_4$.

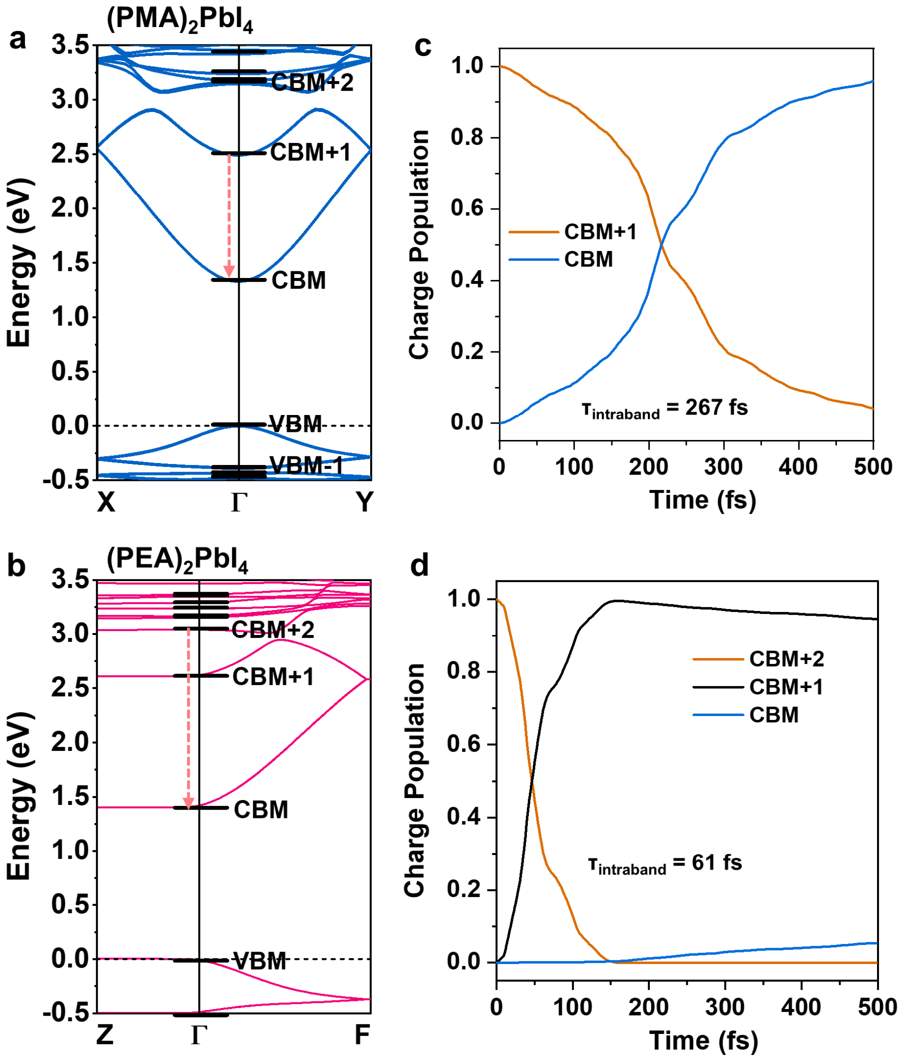

Supplementary Figure 13. Intraband relaxations in 2D RP perovskites. (**a, b**) Electronic bands and energy levels of $(PMA)_2PbI_4$ and $(PEA)_2PbI_4$ at the high-symmetry Γ-point (the VBM is set to zero energy). (**c, d**) Time evolution of hot electron relaxation starting from the CBM+1 for $(PMA)_2PbI_4$ and from the CBM+2 for $(PEA)_2PbI_4$.

Supplementary Table 6. Average electron/hole effective masses of bare electron bands (m^*), optical and static dielectric constants (ε_∞ and ε_S), angular frequencies of a characteristic LO phonon mode (ω, in THz), and electron-phonon coupling constant (α) for $(3AMP)PbI_4$, $(4AMP)PbI_4$, $(PMA)_2PbI_4$, and $(PEA)_2PbI_4$.

**Compound**	$m^*_{electron}$	m^*_{hole}	ε_∞	ε_S	**ω (THz)**	$\alpha_{electron-phonon}$
$(3AMP)PbI_4$	0.182	0.343	4.81	29.86	2.83	2.53
$(4AMP)PbI_4$	0.319	0.808	5.89	20.32	2.92	2.29
$(PMA)_2PbI_4$	0.243	0.697	4.23	22.89	2.64	3.36
$(PEA)_2PbI_4$	0.323	0.558	3.38	20.85	3.38	4.29

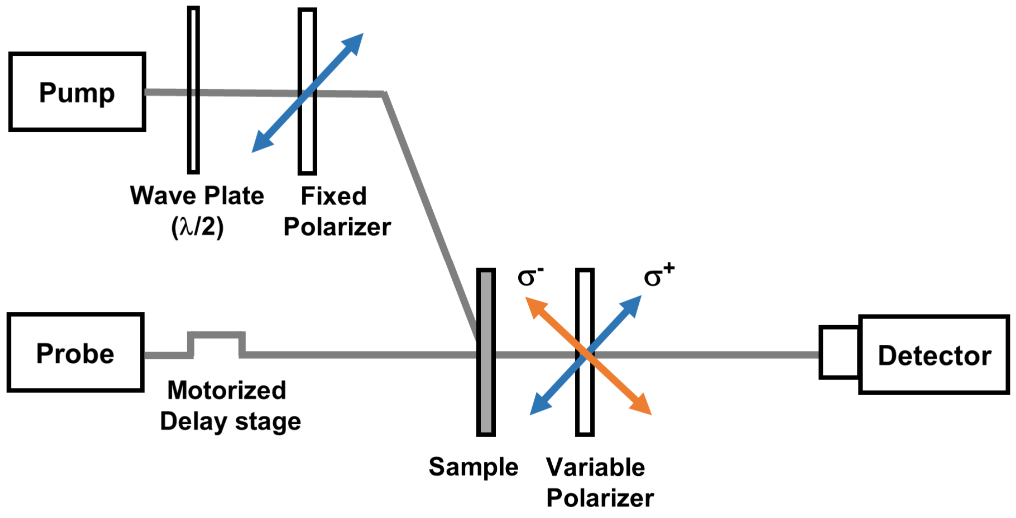

Supplementary Figure 14. Experimental setup used for circular polarized TA measurements. The circular polarized pump pulse is set at an angle of 45° and another circular polarizer is set either parallel or perpendicular to the pump pulse.

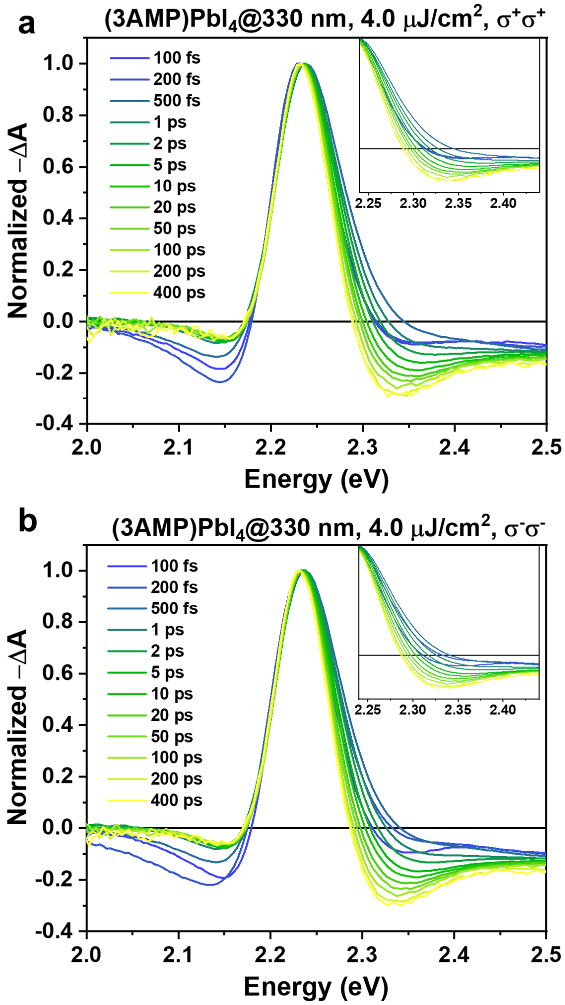

Supplementary Figure 15. Circular polarized TA spectroscopy. Normalized TA spectra of $(3AMP)PbI_4$ by (**a, b**) co-circular polarized pump and probe ($\sigma^+\sigma^+$ and $\sigma^-\sigma^-$) and (**c, d**) counter-circular polarized pump and probe ($\sigma^+\sigma^-$ and $\sigma^-\sigma^+$).

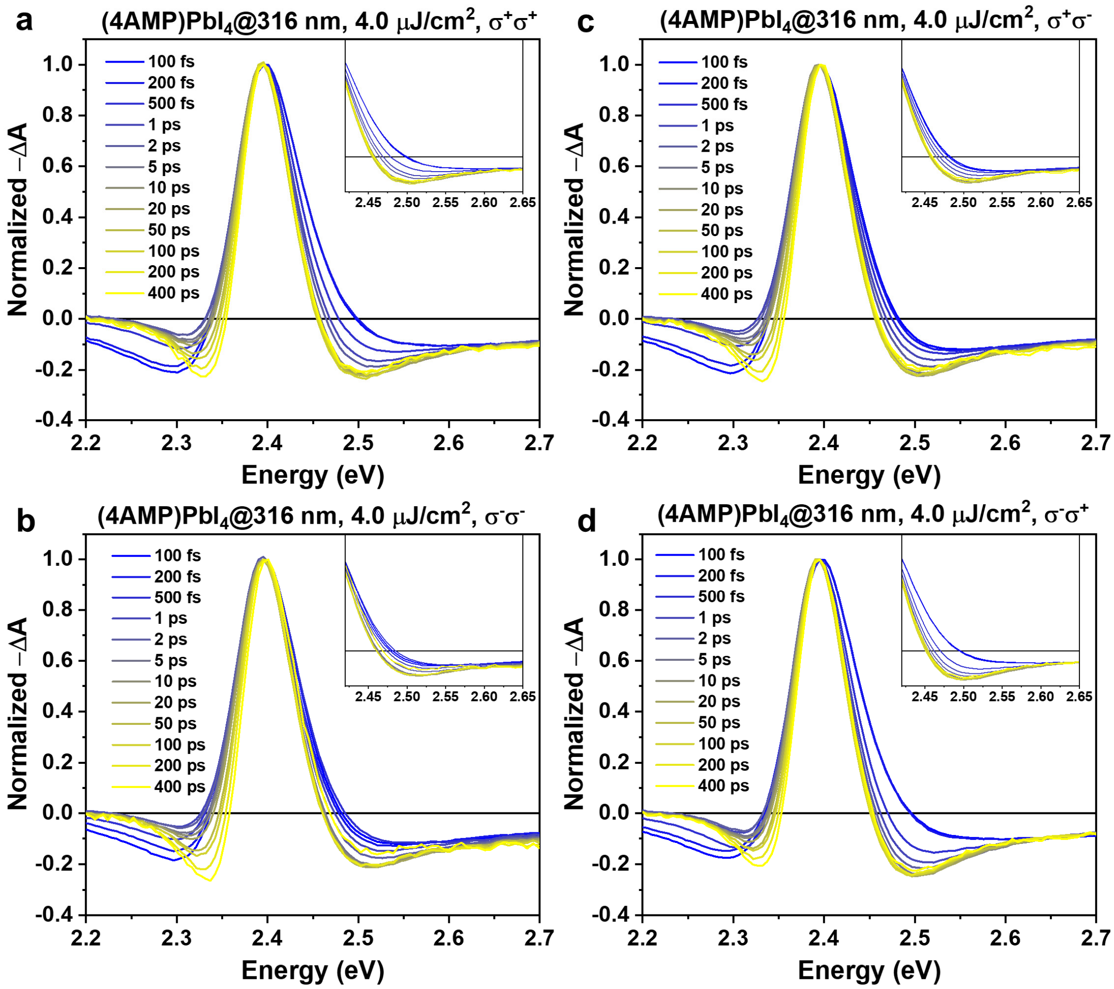

Supplementary Figure 16. Circular polarized TA spectroscopy. Normalized TA spectra of $(4AMP)PbI_4$ by (**a, b**) co-circular polarized pump and probe ($\sigma^+\sigma^+$ and $\sigma^-\sigma^-$) and (**c, d**) counter-circular polarized pump and probe ($\sigma^+\sigma^-$ and $\sigma^-\sigma^+$).

Supplementary Table 7. Fitting parameters of circular polarized transient absorption kinetics probed at 554 nm for the $(3AMP)PbI_4$ film and at 518 nm for the $(4AMP)PbI_4$ film following excitation at 308 nm with a pump fluence of 4 μJ/cm^2.

Film	Pump Type	A_1	τ_1 (ps)	A_2	τ_2 (ps)	A_3	τ_3 (ps)
$(3AMP)PbI_4$	$\sigma^+\sigma^+$	53%	2.10	32%	34	15%	496
	$\sigma^+\sigma^-$	60%	2.50	26%	35	14%	485
	$\sigma^-\sigma^+$	52%	1.85	31%	30	17%	420
	$\sigma^-\sigma^-$	52%	1.63	31%	29	17%	423
$(4AMP)PbI_4$	$\sigma^+\sigma^+$	38%	3.27	38%	62	24%	672
	$\sigma^+\sigma^-$	46%	2.00	33%	30	21%	421
	$\sigma^-\sigma^+$	42%	3.50	38%	72	20%	643
	$\sigma^-\sigma^-$	50%	3.00	30%	46	20%	550

Supplementary References

1. Fu, J. H. *et al.* Hot carrier cooling mechanisms in halide perovskites. *Nat. Commun.* **8**, 1300 (2017).
2. Li, M. J. *et al.* Slow cooling and highly efficient extraction of hot carriers in colloidal perovskite nanocrystals. *Nat. Commun.* **8**, 14350 (2017).
3. Price, M. B. *et al.* Hot-carrier cooling and photoinduced refractive index changes in organic-inorganic lead halide perovskites. *Nat. Commun.* **6**, 8420 (2015).
4. Giannozzi, P. *et al.* Advanced capabilities for materials modelling with quantum espresso. *J. Phys. Condens. Mat.* **29**, 465901 (2017).
5. Umari, P., Mosconi, E., De Angelis, F. Infrared dielectric screening determines the low exciton binding energy of metal-halide perovskites. *J. Phys. Chem. Lett.* **9**, 620-627 (2018).
6. Miyata, K. *et al.* Large polarons in lead halide perovskites. *Sci. Adv.* **3**, e1701217 (2017).
7. Hong, X., Ishihara, T., Nurmikko, A. V. Dielectric confinement effect on excitons in PbI_4-based layered semiconductors. *Phys. Rev. B* **45**, 6961-6964 (1992).
